# Diaphragm Repair with a Novel Cross-Linked Collagen Biomaterial in a Growing Rabbit Model

**DOI:** 10.1371/journal.pone.0132021

**Published:** 2015-07-06

**Authors:** Steffi Mayer, Herbert Decaluwe, Michele Ruol, Stefano Manodoro, Manuel Kramer, Holger Till, Jan Deprest

**Affiliations:** 1 Center for Surgical Technologies and Organ Systems Cluster, Department of Development and Regeneration, Faculty of Medicine, KU Leuven, Leuven, Belgium; 2 Department of Thoracic Surgery, University Hospital Gasthuisberg, KU Leuven, Leuven, Belgium; 3 Department of Pediatric Surgery, University Hospital Padua, Padua, Italy; 4 Department of Obstetrics and Gynecology, University Hospital Monza, Monza, Italy; 5 Department of Radiology, University of Erlangen-Nuremberg, Erlangen, Germany; 6 Department of Pediatric Surgery, University Hospital Leipzig, Leipzig, Germany; 7 Department of Obstetrics and Gynecology, University Hospital Gasthuisberg, KU Leuven, Leuven, Belgium; Institute for Frontier Medical Sciences, Kyoto University, JAPAN

## Abstract

**Background:**

Neonates with congenital diaphragmatic hernia and large defects often require patch closure. Acellular collagen matrices (ACM) have been suggested as an alternative to synthetic durable patches as they are remodeled by the host or could also be used for tissue engineering purposes.

**Materials and Methods:**

2.0x1.0 cm diaphragmatic defects were created in 6-weeks old New-Zealand white rabbits. We compared reconstruction with a purpose-designed cross-linked ACM (Matricel) to 4-layer non-cross-linked small intestinal submucosa (SIS) and a 1-layer synthetic Dual Mesh (Gore-Tex). Unoperated animals or animals undergoing primary closure (4/0 polyglecaprone) served as age-matched controls. 60 (n = 25) resp. 90 (n = 17) days later, animals underwent chest x-ray and obduction for gross examination of explants, scoring of adhesion and inflammatory response. Also, uniaxial tensiometry was done, comparing explants to contralateral native diaphragmatic tissue.

**Results:**

Overall weight nearly doubled from 1,554±242 g at surgery to 2,837±265 g at obduction (+84%). X-rays did show rare elevation of the left diaphragm (SIS = 1, Gore-Tex = 1, unoperated control = 1), but no herniation of abdominal organs. 56% of SIS and 10% of Matricel patches degraded with visceral bulging in four (SIS = 3, Matricel = 1). Adhesion scores were limited: 0.5 (Matricel) to 1 (SIS, Gore-Tex) to the left lung (*p = 0*.*008*) and 2.5 (Gore-Tex), 3 (SIS) and 4 (Matricel) to the liver (*p<0*.*0001*). Tensiometry revealed a reduced bursting strength but normal compliance for SIS. Compliance was reduced in Matricel and Gore-Tex (*p<0*.*01*). Inflammatory response was characterized by a more polymorphonuclear cell (SIS) resp. macrophage (Matricel) type of infiltrate (p<0.05). Fibrosis was similar for all groups, except there was less mature collagen deposited to Gore-Tex implants (p<0.05).

**Conclusions:**

Matricel induced a macrophage-dominated inflammatory response, more adhesions, had appropriate strength but a lesser compliance compared to native tissue. The herein investigated ACM is not a viable option for CDH repair.

## Introduction

In congenital diaphragmatic hernia (CDH) defective development of the diaphragm allows herniation of abdominal organs into the chest. They compete for space during fetal lung development, leading to neonatal ventilatory insufficiency and pulmonary hypertension. The size of the defect varies from a small deficiency of the posterior muscular rim to a complete absence of the diaphragm [1]. According to the CDH registry, neonatal repair of large defects may require insertion of a patch in around 50% of cases, which is a predictor of both, mortality and morbidity [2]. Fetoscopic endoluminal tracheal occlusion (FETO) is an investigational procedure, which aims at inducing lung growth in fetuses with severe pulmonary hypoplasia as documented by prenatal imaging [3]. If the currently ongoing randomized-controlled trials (NCT00763737 and NCT01240057) [4] proof benefit of fetal surgery, a larger number of survivors may need patch repair, as already circumstantiated in initial prospective cohorts operated *in utero* [5,6].

A variety of non-absorbable synthetic, ‘biologic’ as well as composite patches have been used for diaphragmatic repair (reviewed in Gasior et al [7]). Synthetic durable grafts like Gore-Tex (polytetrafluorethylene, PTFE, W.L. Gore and Associates, Flagstaff, AZ, USA) can be considered as the gold standard material for congenital diaphragmatic hernia repair. It is a durable yet non-functional insert between the remnant diaphragmatic borders. As a foreign body, it triggers a process of inflammation, neovascularization and fibrosis but the host tissue does actually not grow into this microporous graft material. Its use may cause thoracic and abdominal wall as well as spinal deformities and detachment of the patch may lead to re-herniation. Other graft related complications are infections, adhesions and short bowel obstruction [8]. To overcome one or more of those limitations, acellular collagen matrix (ACM) patches were considered as an alternative. They may induce a lesser inflammatory response and more organized collagen deposition [9]. Surgisis (Cook Medical, Strombeek-Bever, Belgium) is a porcine non-cross-linked collagen derived from small intestinal submucosa (SIS). During production, cells responsible for the immune response are removed whilst the intact extracellular matrix (collagen type I, III, V) and growth factors (TGF-ß, FGF-2) are conserved. It is degraded in four to 16 weeks by a ‘constructive’ remodeling process that replaces the graft gradually by host connective tissue [10]. In preclinical studies SIS seemed to be an alternative to PTFE [11–13] and in initial small case studies of one, three and ten patients, respectively, no recurrences were observed [7]. However, two larger clinical studies did not find a difference between SIS and PTFE in the rate of recurrences [14,15]. The recurrences after Gore-Tex implantation are thought to be due to the imbalance between the growth of the infant and a permanent material, causing traction and detachment of the prosthesis [16]. Conversely, re-herniations for SIS patches may be related to a rapid degradation of the construct [14]. This prompts the need for alternative ACMs that are resistant to degradation.

Acellular collagen matrices can be produced in large quantities with properties chosen according to condition-specific requirements and application [17]. For diaphragmatic hernia, the ideal matrix would induce a functional repair [18,19] just as other biomaterials successfully do for the reconstruction of the urinary tract and peripheral nerve system [17,20]. Resistance to degradation can be achieved by crosslinking. Pelvicol and its porous version Pelvisoft (C. R. Bard, Inc. Covington, GA, USA) are porcine dermal collagen cross-linked by hexamethylene-di-isocyanate. It has been used experimentally and clinically in pelvic floor surgery, though medium term degradation and graft related complications were observed [21,22]. Therefore, Matricel GmbH (Herzogenrath, Germany) was asked to design an ACM with biomechanical properties as close as possible to that of intact native musculofascial tissue [23] that resists for at least 90 days within a research and development project on tissue engineering for congenital birth defects (EUROSTEC; www.eurostec.eu). We recently tested this ACM for abdominal wall reconstruction in young growing rats [24].

The aim of the present study was to compare this purposely engineered *cross-linked* ACM (Matricel) to the clinical gold standard (Gore-Tex), and the most frequently used, *non-cross-linked* ACM (SIS) for the repair of a surgically created diaphragmatic defect in a reasonably sized, fast growing animal model in order to mimic conditions of neonatal patch repair in CDH.

## Materials and Methods

### Diaphragmatic patches and animals

Three different materials were tested: (1) a 1-mm single layer Gore-Tex Dual Mesh, which consists of expanded PTFE with smooth microporous corrugated macroporous surfaces that was implanted with its rough surface to the thorax; (2) a 4-layer non-cross-linked small intestinal submucosa graft (Surgisis; ‘SIS’); (3) a purpose-designed highly cross-linked ACM (Matricel). Matricel is made of highly purified porcine type I collagen and contains low amounts of other natural fiber forming proteins like elastin and collagen type III. Its *in vitro* and *in vivo* stability was tailored using a non-toxic crosslinking method based on 1-ethyl-3-(3-dimethylaminopropyl)carbodiimide in the presence of N-hydroxysuccinimide. The latter two materials were rehydrated for at least three minutes (min) in sterile saline before implantation. All patches were delivered sterile and originated from the same production lots.

Fifty-four New-Zealand white rabbits were part of this experiment, of which eight served as unoperated controls. For weight consistency, only females were included in the study. Forty-six animals were operated at the age of six weeks, which is the earliest time point of innocuous separation from the mother, at an approximate weight of 1,500 g. At that time, animals were randomly assigned to one of the study groups, defined by the type of surgery or patch used and the time point of sacrifice (60 or 90 days after surgery; Table 1).

**Table 1:** Studied animals and intervention groups.

Group	Initial number	Surgery		Interval	Sacrifice		Final number
	total *n*	weight(g)	deaths *n*	deaths *n*	weight (g)	weight gain (%)	total *n*
**Unoperated control**	**8**	-	-	1	2,907 ± 332	-	**7**
**Primary repair**	**13**	1,547 ± 163	2	3	2,867 ± 279	86 ± 17	**8**
**Gore-Tex**	**11**	1,588 ± 131	-	3	2,842 ± 188	79 ± 10	**8**
**SIS**	**11**	1,600 ± 268	-	2	2,859 ± 273	81 ± 20	**9**
**Matricel**	**11**	1,495 ± 342	-	1	2,741 ± 279	89 ± 26	**10**
**Total *n***	**54**		**2**	**10**			**42**
*overall p-value*		*0*.*8*			*0*.*8*	*0*.*8*	

Values presented as absolute numbers per group (n) or mean ± SD with overall p-value (ANOVA).

### Interventions

Perioperative antibiotic prophylaxis consisted of Penicillin G (Continental Pharma, Brussels, Belgium; 50.000 IE/kg body weight (BW)/day intramuscular (IM)), continued for three days. Animals were premedicated with ketamine (Ketamine 1000 CEVA; Ceva Sante, Brussels, Belgium; 35 mg/kg BW IM), xylazine (Vexylan; Ceva Sante; 5mg/kg BW IM) and buprenorphine (Vetergesic; Ecuphar, Oostkamp, Belgium; 0.05 mg/kg BW IM) for sedation, anxiolysis and pain relief. Animals were pre-oxygenated using a facemask followed by an induction dose of inhaled isoflurane (IsobaVet; Schering-Plough, Kenilworth, NJ, USA) 0.5% in 1.5 L/min oxygen. Animals were intubated with endoscopic assistance using a rigid neonatal bronchoscope (10339F) with a 1.3 mm fibre endoscope (11540AA; both Karl Storz Endoskope, Tuttlingen, Germany) to insert a semi-rigid guide wire (TSF-35-145, 0.35", 145 cm; Cook Medical) over which a 10–14 Ch endotracheal tube was advanced, according to the size of the animal. Animals were allowed to breath spontaneously and in case of desaturation they were ventilated using a 0.5 L ambu bag-valve-mask (Dräger, Lübeck, Germany). Anesthesia was maintained by isoflurane 0.5 to 1.5% in 1.5 to 2.0 L/min oxygen.

**Fig 1:**
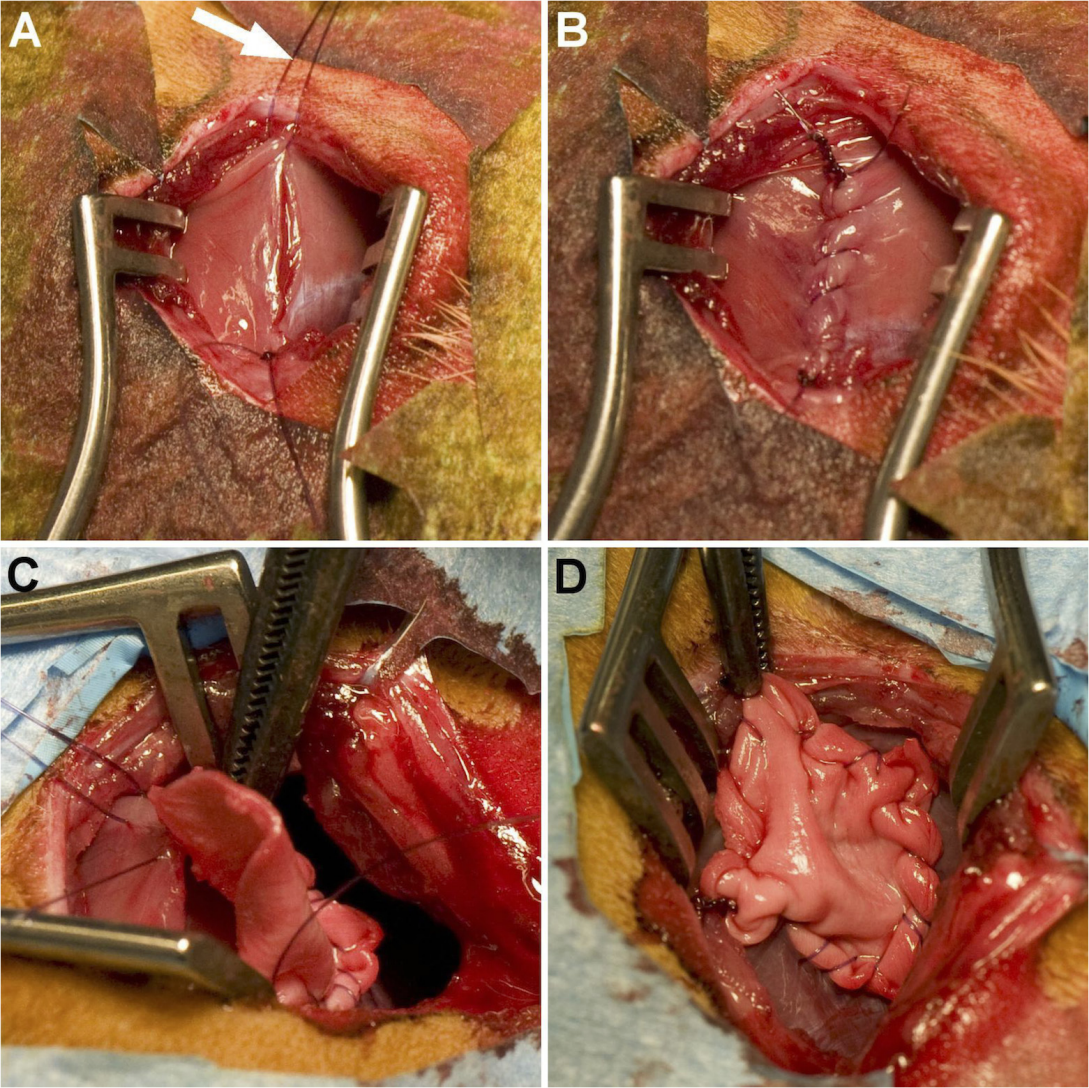
Surgical procedure. A left-dorsolateral thoracotomy was performed to expose the diaphragm. After placement of a traction suture (A, *arrow*) a 2.0 x 1.0 cm defect was cut in the tendomuscular part of the diaphragm and repaired by either placing a running suture (B) or inserting a cone-shaped patch (C, D) made from different materials.

All surgical interventions were performed under sterile conditions as well as continuous monitoring of the heart rate and oxygen saturation. The animal was placed on its right side; the left laterodorsal thorax and upper abdomen were shaved, disinfected with povidone iodine (Isobetadine; Astra Medica, Brussels, Belgium) and covered with sterile draping. A left anterolateral thoracotomy [5] was performed in the third lowest intercostal space and a rib spreader was inserted to allow access to the diaphragm (Fig 1). A 5/0 polypropylene traction suture (Prolene; Ethicon, Dilbeek, Belgium) was placed at the anterolateral edge of the diaphragm prior to standardized resection of 2.0 x 1.0 cm of the lateral tendomuscular part of the diaphragm, approximating ⅓ to ½ of the diaphragm. This defect was either primarily repaired by a running 4/0 polyglecaprone suture (Monocryl; Ethicon) or overlaid by a 2.0 x 2.0 cm cone-shaped patch—as clinically done [25]—allowing an increased abdominal capacity. We temporarily left a thoracic drain (CH 8–10, Kendall Argyle suction catheter; Covidien, Belgium) connected to a ‘water-seal’ system until the thoracic wall was closed in layers using 4/0 polyglactine (Vicryl; Ethicon). Once the perioperative pneumothorax was evacuated, the drain was removed. The skin was closed with a running intracutaneous polyglecaprone suture (3/0 Monocryl; Ethicon). The wound was disinfected again with povidone iodine and covered with aluminum spray to prevent scratching and biting during the reconvalescence period.

### Ethics Statement

All animals were housed separately at the Animalium of the Faculty of Medicine of the Katholieke Universiteit Leuven, Belgium. They were allowed chow and water ad libitum throughout the experiment and were examined postoperatively on a regular basis and all efforts were made to minimize suffering. This study was carried out in strict accordance with the recommendations in the Guide for the Care and Use of Laboratory Animals of the National Institutes of Health. The protocol was approved by the Ethical Committee of the Katholieke Universiteit Leuven, Belgium (Permit Number: P163-2010).

### Radiography

Prior to sacrifice, animals were sedated with ketamine and xylazine to document lung collapse, displacement of viscera or skeletal deformation by x-ray, if any (Embrace DM 1000 Mammography System; Agfa-Gevaert, Mortsel, Belgium; collimation size 24 x 29 cm, thickness 280 mm, voltage 28 kVp, engine load 65 and 80 mAs, each in two planes). X-rays were taken by one investigator (SMay) and analyzed on a picture archiving and communications system (PACS) (Impax, Agfa-Gevaert) by an experienced radiologist (MK), who was blinded to the study groups. Any signs of infection, atelectasis, deformation of the spine or the thoracic cage as well as elevation of or herniation through the diaphragm were noted.

### Sacrifice

Animals underwent euthanasia by intravenous injection of 1 mL T61 (embutramide 200 mg, mebezonium 50 mg and tetracaine hydrochloride 5 mg; Hoechst Marion Roussel, Brussels, Belgium). The thoracic scar was examined for signs of infection and dehiscence before a midline laparotomy was performed. The abdominal cavity was opened and inspected for the presence of a diaphragmatic defect or bulging, herniation of abdominal organs, fluid collections, infection and adhesions. The severity of adhesions was quantified by documenting the density (grade 0–4) at the site of incision (lateral thoracic wall) and repair (to the liver and left lung) as well as remote from the surgical field (dorsal thoracic cage) [26]. Subsequently, the diaphragm was explanted en bloc. All findings were documented photographically. The left diaphragm was cut in an anterior-posterior direction at the mesh-tissue interface into a strip of 1 cm in width and at least 2 cm in length used for tensiometry and a 0.5 x 1.0 cm piece for histology. For internal control a similarly sized muscular ‘mirror’ strip of the right diaphragm was used. Explants for tensiometry were kept in normal saline solution at room temperature until testing, which was at the latest 2 h after euthanasia.

### Tensiometry

Prior to biomechanical testing, the width, length and thickness of each explant were assessed using a digital micrometer (Mitutoyo, Japan, accuracy 0.01 mm) and averaged. Tensiometry provides information on the visco-elastic properties and the resistance to disruption of the tested tissue. Biologic materials have a complex non-linear stress-strain behavior that is defined by two regions: (1) the ‘comfort’ zone in which physiological forces apply, showing a compliant response with large strain at low stress; (2) the ‘stress’ zone in which higher resistance to deformation is displayed, leading to a rapid rise of stress for increasing deformation [27]. We used a 500N Zwick uniaxial tensiometer with a 200-N load cell and Textxpert II software (Zwick GmbH & Co. KG, Ulm, Germany). Samples were inserted tension-free at the level of the native diaphragmatic tissue resp. explant between the clamps at a grip-to-grip separation of 1.5 cm and tested until failure. All tests were performed at an elongation rate of 60 mm/min. The length (mm) and the corresponding force (Newton; N) were recorded. The stress (N/mm^2^) needed for a certain elongation divided by the non-deformed cross-section of the tested sample (thickness x 1.0 cm width) was calculated. The compliance (N/mm^2^) in each zone was determined as regression line between two pre-defined points. Also, the maximal stress (N/mm^2^) at the point of disruption as well as the location of breakage (native tissue, interface or implant area) was documented.

### Histology and immunohistochemistry

Specimens were fixed in 10% formalin overnight, embedded in paraffin and cut in 4 μm slices. For all outcome measures, five fields per slide randomly selected from the same region, i.e. at the interface between implant and surrounding tissue, were scored at a magnification of 400x using an Axioskop 40 microscope (Carl Zeiss, Oberkochen, Germany) by one operator (MR) who was blinded to the study groups and outcome, and subsequently averaged. A hematoxylin and eosin staining was performed to quantify the presence of foreign body giant cells (FBGC), polymorphonuclear cells (PMN) and neoangiogenesis. A semi-quantitative scale was applied that accounted for 0 (0), 1–5 (1), 6–9 (2) and ≥10 (3) cells as well as 0 (0), 1–3 (1), 4–9 (2) and ≥10 (3) number of vessels including edge-gated ones without any distinction in size resp. muscularization [28].

The number of macrophages was determined using a selective monoclonal mouse anti-rabbit RAM-11 antibody (MO63; Dako Corp., Carpinteria, CA, USA) [29]. In brief, after blocking endogenous alkaline phosphatase with HCl 0.2 M for 10 min and blocking unspecific binding with blocking buffer (bovine serum albumin 2%, non-fat dry milk 1%, Tween 80 0.1%) for one hour at room temperature, slides were incubated with RAM-11 1:50 over night at 4°C followed by incubation with goat anti-mouse secondary antibody (1:50; Dako Corp.) and normal rabbit serum (1:25; Dako Corp.) for 30 min at room temperature and final counterstaining with Methyl Green 2% solution for 30 min. The amount of RAM-11 positive cells was determined in absolute numbers.

The organization, composition and amount of collagen were assessed semi-quantitatively using Movat stain [30]. Collagen organization was scored from totally disorganized to a well-organized scar tissue (0–3), its composition from absent (0), cellular (1), mixed (2) to a (nearly) acellular (3) scar and its amount from absent (0), minimal (1), moderate (2) to abundant (3) [28].

### Statistical analysis

Statistical analysis was performed using JMP 9 (SAS Institute, Cary, NC) and results were plotted by Prism 5 (GraphPad Software Inc, La Jolla, CA, USA) software. Dichotomous and ordinal measurements (adhesion score) were analyzed on basis of contingency tables by Fisher’s exact test or Chi-Square test. Continuous data were tested for normal distribution using a Goodness-Of-Fit test (Shapiro-Wilk). Normally distributed data (weight at surgery and harvest, maximal stress at disruption, collagen amount) was further analyzed applying a two-sided t-test for two-group comparison or a one-way analysis of variance (ANOVA) with posthoc Tukey test for multiple-group comparison. For non-parametric data, a Mann-Whitney test for two-group comparison or a Kruskal-Wallis test with posthoc Wilcoxon each pair test for multiple-group comparison was applied. Matched pairs analysis (paired t-test or Wilcoxon Signed Rank test) was performed for within-subject comparisons.

The relationships between multiple variables were assessed by regression analyses. After implementation of an effect screen for ‘study group’, ‘time point of sacrifice’ and ‘presence of bulging’ applicable effects were selected to provide the best-fitting model. If necessary, data was transformed into normal distribution using the square root, square, log, inverse or box-cox transformation. Analysis of best-fitting models did not reveal an impact of the time point of sacrifice on the assessed outcome measures. Consequently, all outcome measures from both time points were pooled, resulting in a sample sizes of minimum seven and up to 10 per group (Table 1). Significance level was set at p < 0.05. Data are presented as mean ± standard deviation (SD) if not indicated differently.

## Results

### Survival and gross anatomical findings

Two animals died during surgery (both had a primary repair) and ten within the first two weeks after, equally distributed among groups (Table 1). We were unable to determine their direct cause of death. Forty-two of 54 animals survived until sacrifice 60 (n = 25) resp. 90 (n = 17) days after surgery and were analyzed. Their overall weight was 1,554 ± 242 g at surgery and 2,837 ± 265 g at obduction, hence with an overall weight gain of 84 ± 19%, which corresponds to nearly a doubling in size.

**Fig 2:**
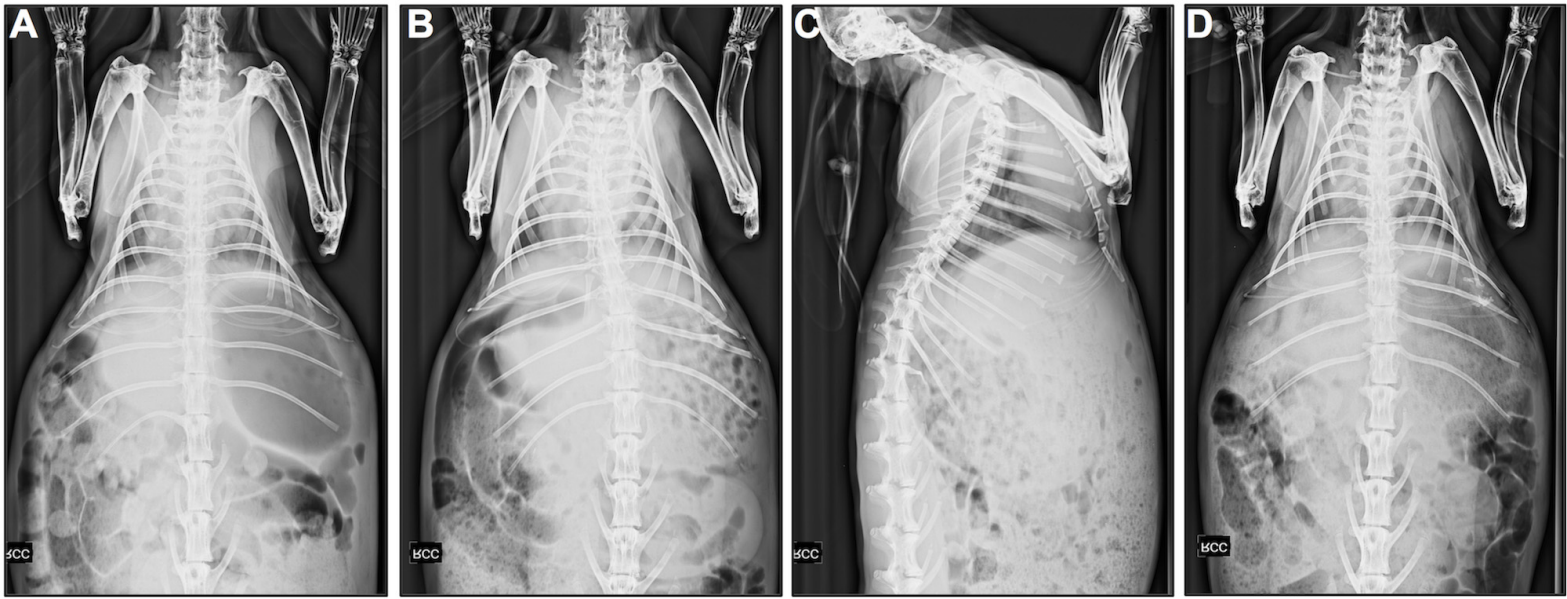
Radiographic evaluation. Unoperated control (d90; A), left-convex scoliosis, postoperative changes of the 9^th^ and 10^th^ left rib (SIS d90; B), discrete elevation of the left diaphragm (SIS d60; C), and discrete reduced left-thoracic volume with postoperative calcifications adjacent to the 8^th^ left rib (Matricel d90; D).

Only limited changes were observed on chest x-ray. Discrete elevation of the left diaphragm was seen in three animals at 60 days, one in the Gore-Tex and SIS group (Fig 2C) and remarkably also in an unoperated control. We did not find true herniation of abdominal organs through the diaphragm nor noticed any signs of infection or pulmonary atelectasis. One animal presented with a left-convex scoliosis (Fig 2B; SIS d90) and one (Matricel d90) had a slightly reduced left-thoracic volume with postoperative calcifications adjacent to the 8^th^ left rib (Fig 2D).

**Fig 3:**
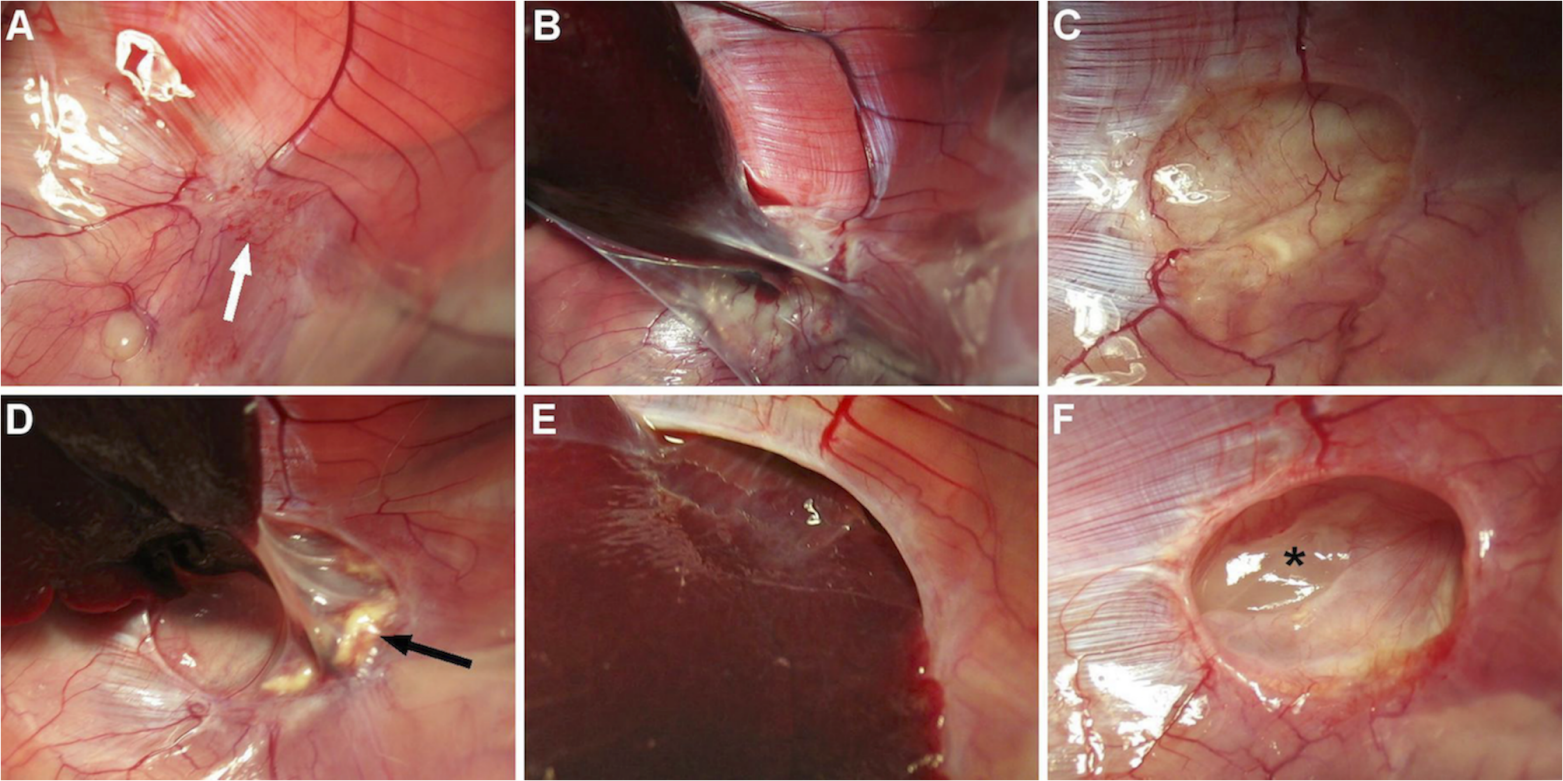
Macroscopic findings. A. Primary repair (d60), polyglecaprone suture absorbed (*arrow*). B. Gore-Tex (d 60), encapsulated with perihepatic adhesions. Matricel patches were preserved in all (d60; C) but one animal (d90; D, *arrow =* patch remnant) with high-grade perihepatic adhesions (D). SIS patches were degraded in five animals with bulging of viscera in three (d60, E). Seroma was observed twice (d 60, F, *asterisk*).

The thoracic wall scars were unremarkable, neither were there intrathoracic signs of infection and none of the diaphragmatic repairs failed. Adhesions to the side of the thoracic incision were comparable in all operated groups (median grade 2; *p = 0*.*8*). Adhesions away from the surgical field were sparse (median grade 0) and only present after patch repair (*p = 0*.*2*). However, adhesions to the left lung (*p = 0*.*008*) and the liver (*p<0*.*0001*) differed between groups. The primarily repaired animals had no adhesions to the liver or lung (Fig 3A). Adhesions to the left lung were of a median grade 0.5 in Matricel animals and of a median grade 1.0 in SIS resp. Gore-Tex, whilst hepatic adhesions ranged from median grade 2.5 in the Gore-Tex group and grade 3.0 in the SIS group to a grade 4.0 in the Matricel group (Fig 3B and 3D).

Gore-Tex patches appeared virtually unchanged, with conservation of their conical shape, yet encapsulated. Matricel patches were partly degraded by the host (Fig 3C). However, in one animal at 90 d there was no recognizable patch material anymore, with liver bulging into the thorax (Fig 3D). For SIS repaired animals, the patch was completely degraded in five animals. Of those, three showed bulging of the viscera into the thorax (Fig 3E), which was not observed in the case of the material being still recognizable. Two implants had serous fluid accumulation between layers (‘seroma’; Fig 3F). If the patch had vanished (SIS [n = 5], Matricel [n = 1]) the replacing scar appeared to be a thin but vascularized connective tissue layer (Fig 3D–3F), with bulging liver in three (Matricel [n = 1], SIS [n = 2]) and bulging of liver plus stomach in one animal of the SIS group. The weight increase in the animals with degraded patches was not different from those without (SIS; *p = 0*.*8*).

### Tensiometry

We first compared the strength and compliance of left and right-sided native diaphragmatic strips in unoperated controls. These were not different. Also, the untouched right-sided diaphragmatic strips of all operated animals had similar biomechanical properties and did not differ from unoperated controls *(data not shown)*. These findings excluded side differences as well as an impact of surgery itself on the biomechanical properties of the diaphragm. Thus, we were able to perform paired comparisons within each animal between the implanted construct (left side) and the respective untouched diaphragmatic tissue (right side) followed by in-between groups comparison.

**Fig 4:**
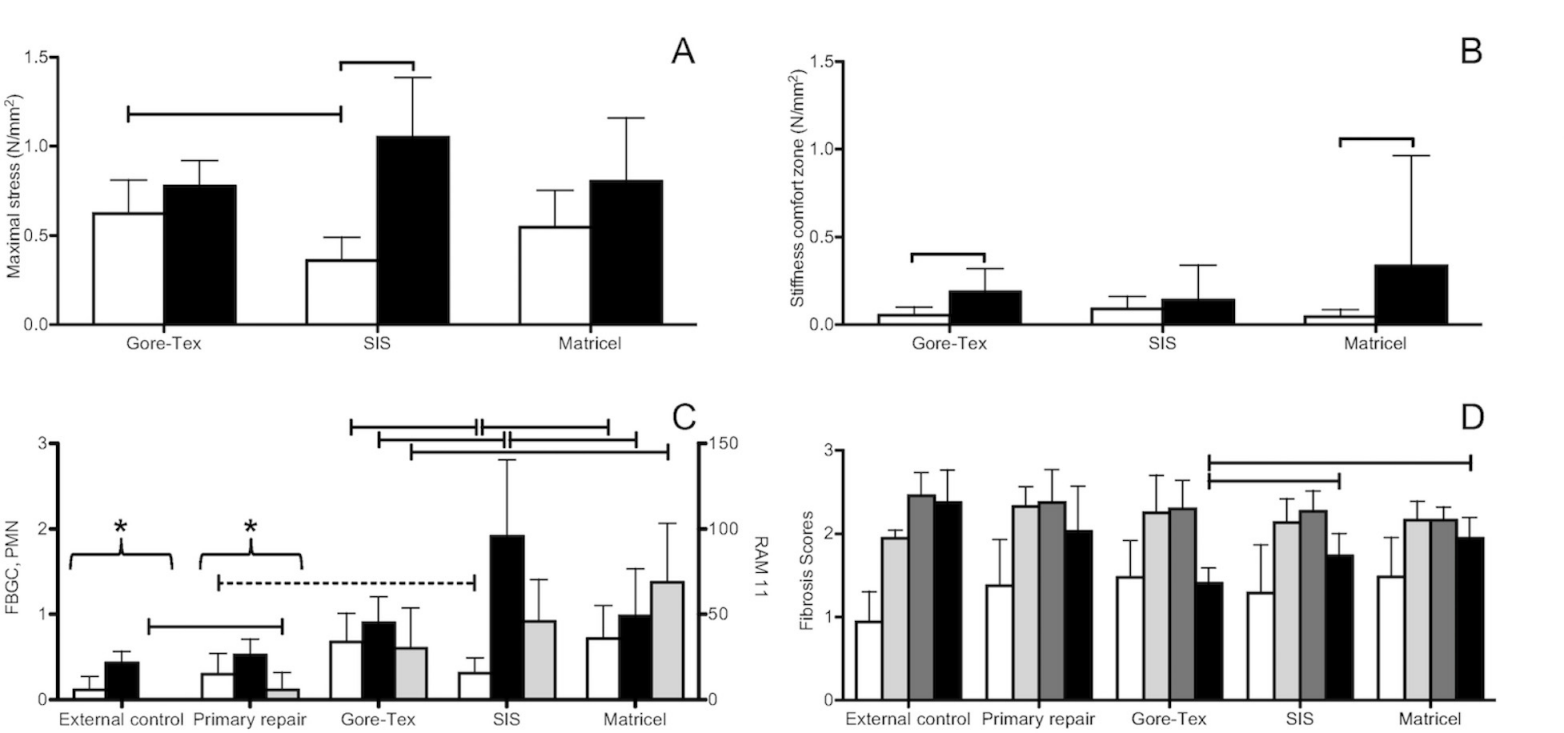
Tensiometric and histological results. Maximal stress at the point of disruption (A, *p = 0*.*01* ^$^) and stiffness of the comfort zone (B, *p = 0*.*3*) for the mesh-tissue interface *(white)* compared to the right-sided unoperated diaphragm *(black)*. Inflammatory response (C): foreign body giant cells (FBGC; *p = 0*.*0008; white*), polymorphonuclear cells (PMN; *p<0*.*0001; black*) resp. macrophages (RAM 11; *p<0*.*0001; light grey*). Fibrotic process (D): collagen organization (*p = 0*.*2; dark grey*), composition (*p = 0*.*0005; black*) and amount (*p = 0*.*1* ^$^; *light grey*), neovascularization (*p = 0*.*2; white*). Mean+SD with overall p-value (Kruskal-Wallis test or ANOVA [^$^]). Multiple groups comparison (Wilcoxon each pair test or posthoc Tukey test): solid line = significant, dashed line = non-significant, * = significant difference between unoperated controls / primary repair and the three patch materials. Matched-pairs comparison (paired t-test or Wilcoxon signed rank test): [squared bracket = significance level].

The point of disruption was at the interface between the implant and the host tissue in 24 of 27 samples. In only one (Gore-Tex) the explant and in two (Matricel) the native tissue disrupted first. The maximal strength at disruption (Fig 4A) of SIS explants was lower than that of the right-sighted native tissue (*p<0*.*0001*), irrespective of the visible persistence (*p = 0*.*01*) or absorption (*p = 0*.*005*) of the initial material. It was also lower than that of Gore-Tex (*p = 0*.*01*) but not of Matricel patches (*p = 0*.*08*). Conversely, the maximal strength at disruption of Matricel and Gore-Tex explants fell in the range of what was measured in the intact native tissue (both *p = 0*.*08*). There was neither any difference between the two materials (*p = 0*.*6*).

The second parameter measured was the compliance of the explant within the ‘comfort’ zone (Fig 4B). This is the low force and displacement range, which is believed to be within the physiologic range [27]. Matricel (*p = 0*.*01*) and Gore-Tex (*p = 0*.*008*) but not SIS (*p = 0*.*6*) explants were less compliant (i.e. stiffer) than native tissue. Though probably clinically less relevant, compliance in the ‘stress-zone’ was also determined and turned out to be significantly lower than that of native tissue for all patch materials *(data not shown)*.

### Microscopy

**Fig 5:**
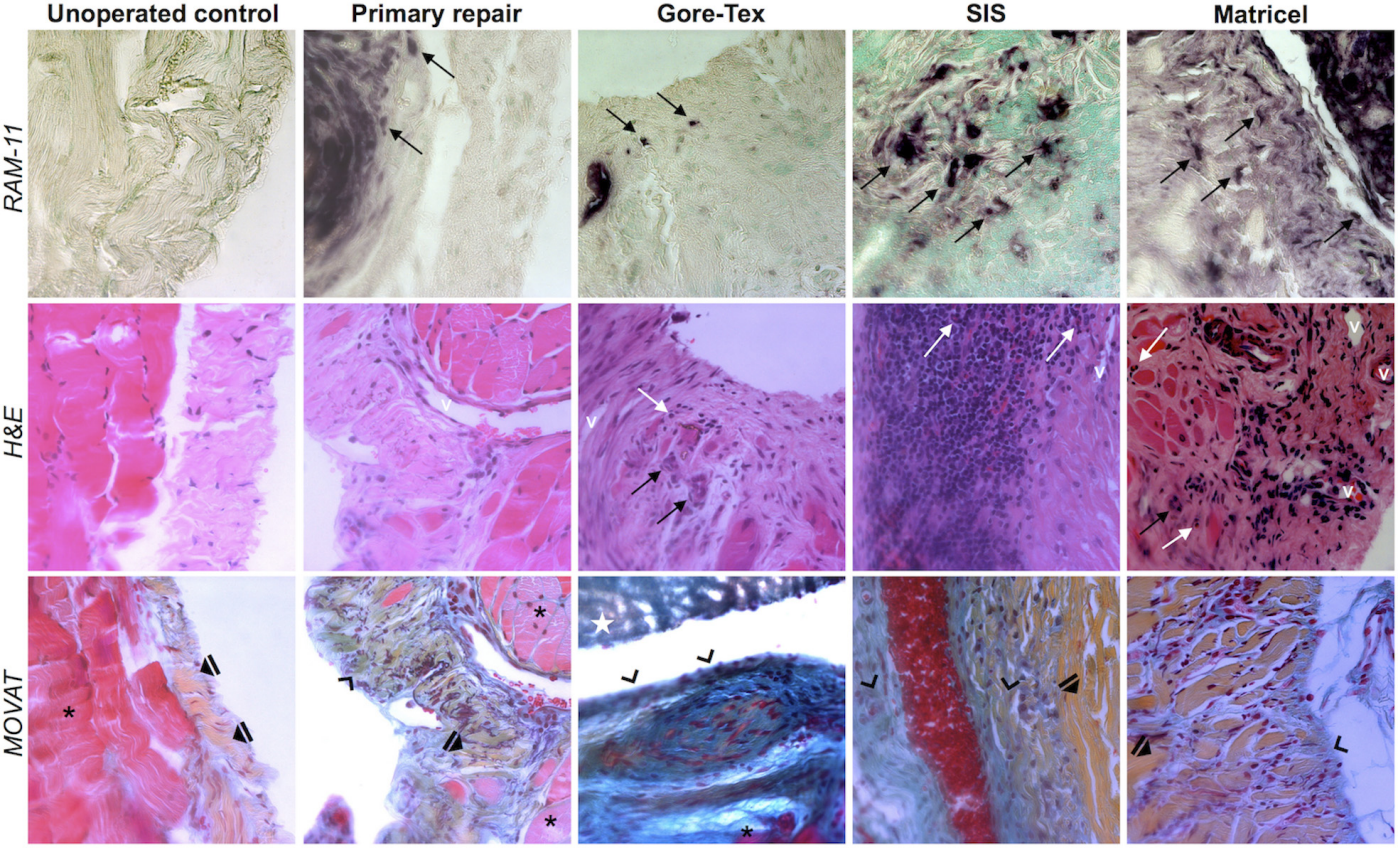
Histology. Representative micrographs of all groups (columns) obtained at x400 magnification with identical settings for each stain from the mesh-tissue interface. Meshes are invisible form degradation or processing, respectively. Inflammation is represented by the number of macrophages (first row, black arrows), foreign body giant cells (second row, black arrows) and polymorphonuclear cells (second row, white arrows), fibrosis depicted by the amount of neovascularization (second row, v vessel) as well as the amount and composition of collagen (third row, * muscle, ⏏ mature collagen, ⌃ immature collagen, ★ patch). H&E: Hematoxylin-eosin.

The overall inflammatory response (Fig 4C and Fig 5) was higher in patched groups as compared to primary repairs. Gore-Tex explants showed moderate levels of inflammation. Matricel induced higher numbers of macrophages in comparison to Gore-Tex (stained by RAM-11; *p = 0*.*01*). In the SIS group, there were fewer foreign body giant cells yet more polymorphonuclear cells as compared to Gore-Tex (*p = 0*.*01*; *p = 0*.*04*) and Matricel (*p = 0*.*009*; *p<0*.*05*). The fibrotic response did not differ between groups (Fig 4D), in other words, the degree of neo-angiogenesis as well as the amount and organization of collagen deposited by the host were comparable. However, Gore-Tex was surrounded by less mature collagen as compared to SIS (*p = 0*.*01*) and Matricel (*p = 0*.*0007*). When considering SIS explants with or without material remnants, we could not detect a measurable difference in inflammatory or fibrotic scores, except that there were less polymorphonuclear cells when SIS was already absorbed (*p = 0*.*04*).

## Discussion

We aimed to compare the performance of a purpose-designed ACM (Matricel) for diaphragmatic reconstruction with clinically used materials (Gore-Tex and SIS) in a fast growing animal model. Therefore, we closed surgically induced diaphragmatic defects in six weeks-old rabbits, which was associated with a perioperative mortality of 22%. The remaining animals were allowed to survive two resp. three months by which time they nearly doubled in size. In none the repair formally failed, however, there were remarkable differences between materials.

The use of SIS was associated with seroma formation in 22%, visible disappearance of the patch in 56%, and apparent bulging on obduction in 33%. On x-ray however, that bulging was *not* visible. SIS explants also had a lower bursting strength, but a compliance in the comfort zone equivalent to that of native tissue. Microscopic examination of the visibly thin scarred area revealed a prominence of polymorphonuclear cells and a milder foreign body reaction than in Gore-Tex resp. Matricel implanted animals. Surprisingly, despite cross-linking, which should make the implant resistant to collagenase activity hence degradation, the purpose-designed novel ACM Matricel degraded in one out of ten animals. In those, it also led to bulging (though again *not* visible on x-ray). Matricel had a bursting strength within the range of native tissue but a lower compliance. Microscopically, there was a predominance of macrophages and a comparable amount of foreign body giant cells as compared to Gore-Tex implants. Gore-Tex preserved its initial conical shape and induced encapsulation with more foreign body giant cells than SIS and less mature collagen as to ACMs. Though strong, Gore-Tex explants were less compliant than native tissue.

Irrespective of the clinical indication for implants, in view of patient safety, preclinical experimentation is an important step of the introduction cycle of novel products [31]. Considering the shortcomings of current synthetic durable grafts for diaphragmatic reconstruction, our industrial partner engineered a new ACM, which we asked to withstand degradation for more than three months, until we anticipated a proper scar would be generated. For that purpose, a highly cross-linked ACM was designed, which we studied in a reasonably sized animal model mimicking the effect of fast growth of infancy.

In view of this, we think our study has a number of strengths. Rabbits weighed on average 1,500 g at the operation and almost doubled in size until sacrifice. This was up to three months later, a time period that equals easily 24 months in humans. Clinically, most recurrences are observed within the first 12 to 18 months of life so that the used observation period may be representative [15,32]. Though there was a fairly high loss rate (22%), we had a reasonable sample size at the time of harvesting. The testing methods were comprehensive and met the standard of evaluation of implants for abdominal wall as well as pelvic floor reconstruction [23,33]. Our results reproduce clinical observations for SIS patches in diaphragmatic repair, suggesting that this model is representative [14].

However, we acknowledge a number of shortcomings of the present study. In view of future experiments, knowing the exact cause of death would have been informative. On the other hand, the fact that also one control animal died demonstrates that not all the risks are from the surgical reconstruction. We did not include longitudinal follow up measurements, e.g. by non-invasive testing (such as chest x-ray) nor did we perform functional tests, such as in vivo mobility or contractility. In retrospect, the study period might still have been too short to assess later surgical failures or to study the long-term fate of cross-linked ACM, as earlier done in a two-year follow up study on abdominal wall reconstruction in rabbits [21]. In that experiment cross-linked Pelvicol was used and late onset (180 d) degradation as well as late graft-related complications like calcification of the material were observed. Though comprehensive histologic evaluation was done, most morphometric methods remain semi-quantitative and with uncertain functional correlate. This limitation is common in this type of surgical experiments, especially in rabbits, which have a different collagen metabolism. Thus, caution is advised when extrapolating ACM degradation [21]. Rats are an interesting alternative, which also allows for sophisticated and validated molecular testing [34]. Conversely, rats are not representative in size and long-term experiments may be more problematic. Finally, biomechanical performances have been assessed by uniaxial tensiometry, which does not account for the three-dimensional loading forces that are present in the body. Therefore, results should be transferred to clinical practice with care [35].

Apart from those methodological considerations, we did a number of interesting observations. Again, implantation of SIS confirmed earlier clinical observations, such as fluid accumulation between layers. This is thought to be due to the layered synthesis and slow and delayed incorporation of the inner layers of the patch material [36]. We also observed bulging, but that was only *ex vivo*. *In vivo* a normal position of the reconstructed diaphragm was documented radiographically and the animals were clinically healthy. Thus, its functional relevance remains doubtful. Though the strength of the diaphragm after partial or full degradation of SIS was weaker than that of native tissue, true dehiscence or frank ‘recurrence’ did not happen in our animals. Hypothetically, SIS may be associated with a higher *early* recurrence rate due to the loss of structural integrity if the patch dissolves before proper tissue ingrowth [15,37]. But in clinical practice recurrences have been described as occurring at various time points following initial repair [14]. In our study, degradation was also yet less frequently (n = 1) observed with the cross-linked Matricel ACM. We have seen that variability in earlier experiments with other cross-linked materials as well as clinically [21,38]. In general, cross-linking is used to make collagen matrices resistant to endogenous collagenase activity. Several cross-linkers and more advanced degrees of cross-linking may influence the ultimate resistance to degradation. We asked the manufacturer to produce a prototype candidate ACM with increasing degrees of cross-linking to make the material resistant to degradation within the duration of the experiment. However, several animal experiments and clinical experience show that despite cross-linking, degradation, but also other inflammatory changes, such as stiffening, and even calcification do occur [21,39,40]. The consequence of degradation is that the replacing tissue may not be able to prevent bulging. Accordingly, bulging was observed more often for non-cross-linked SIS than cross-linked Matricel.

In our experiment the grade of adhesions was different according to the nature of the implant. Gore-Tex induced adhesions to the lung and to the liver, although the smooth side of the Dual Mesh faced the abdominal cavity to prevent the latter [41,42]. However, perihepatic adhesions were more pronounced for ACM. Manifest perihepatic adhesions might reduce the chance for recurrence and induce the regeneration of the artificial diaphragm by providing sufficient blood supply, though they are not a desired effect [12,37]. Interestingly, whatever diaphragmatic reconstruction method has been used in the presented study, few to no bowel adhesions were observed. This is of particular interest because adhesive small bowel obstruction after CDH repair has been clinically reported in a considerable number of cases, irrespective of the type of surgery [43,44]. The paucity of intestinal adhesions in our study could be due to the use of polyglecaprone sutures because of its anti-inflammatory properties [45] and the surgical access of thoracotomy, which is rarely used clinically [5,8]. At the same time adhesions to the lung were limited and of low-grade for all patch materials, which is claimed to be due to the anti-adhesive phospholipids of lung surfactant and the presence of mesothelial cells [37,46].

A number of clinical conclusions might be drawn from this experiment. At first, the ‘standard of care’ product Gore-Tex may certainly do from a surgical viewpoint, as its strength is unquestioned. However, it impedes appropriate incorporation into the host with limited collagen maturation, it is associated with significant adhesions, and the construct does not have the compliance of native tissue. On the other hand, the herein used ACM bio-implants seem to be reassuring when looking at the inflammatory reaction. However, bulging of non-cross-linked SIS was observed after sacrifice and its strength was reduced. The Matricel prototype ACM degraded in one animal. It provoked more perihepatic adhesions, of which the clinical relevance is difficult to assess. Moreover, Matricel as well as Gore-Tex patches had a decreased compliance despite an appropriate bursting strength as compared to normal tissue. However, strength may be one worry; another one is the quality of the newly generated tissue. SIS explants were the only ones to have compliance comparable to that of native tissue. These findings are in line with the results assessed in a rat-model for musculofascial reconstruction of abdominal wall defects for cross-linked Matricel ACM in comparison to cross-linked Pelvisoft patches [24]. Although all implants were well tolerated and effective during the 90 days period, passive biomechanical properties of explant were not comparable to that of native tissues.

Beyond the presented research into novel materials for patch repair in CDH, ACMs also play a key in tissue engineering. Ideally, after a diaphragmatic defect has been diagnosed prenatally, amniotic fluid stem cells are obtained e.g. at amniocentesis, expanded and engineered to repair the defect with autologous tissue readily available for surgical correction after birth (reviewed in [47,48]). By then, PTFE remains the gold standard for CDH closure. But with further advancements in stem cell biology and in line with the rapid growth of the tissue-engineering field, diaphragmatic engineering may find its way into clinics in the foreseeable future.

## Conclusions

Herein we described the surgical reconstruction of the diaphragm in six weeks-old rabbits that nearly doubled in size and reached a near adulthood weight by the time they were harvested. More than half of the SIS implants degraded with apparent bulging in 60%. Those explants had a normal compliance though low bursting strength. Cross-linked Matricel ACM implants degraded in one animal; its bursting strength and compliance were comparable to that of Gore-Tex reconstructions. Both materials were less compliant than normal tissue. Additional longer-term studies and design of alternative acellular matrices seem recommendable prior to consideration for clinical use.
